# Multicenter study results of the med-el partial ossicular replacement prostheses

**DOI:** 10.1007/s00405-025-09799-7

**Published:** 2025-11-16

**Authors:** Susan Arndt, Christian Offergeld, Wendelin Wolfram, Lisa Niederwanger, Dirk Beutner, Nicholas Bevis, Joachim Hornung, Lava Taha, Piotr H. Skarżyński, Łukasz Plichta, Thomas Lenarz, Susan Busch, Esther Schimanski, Georg Sprinzl, Astrid Magele, Benjamin Loader, Franz Windisch, Nina Rubicz, Paul Martin Zwittag, Dominik Riss, Christoph Arnoldner

**Affiliations:** 1 Department of Otorhinolaryngology-Head and Neck Surgery, Faculty of Medicine, Medical Center-University of Freiburg, University of Freiburg, 79106 Freiburg, Germany; 2 Department of Otorhinolaryngology, Klinikum Wels-Grieskirchen, Wels, 4600 Austria; 3 Department of Otolaryngology, University of Goettingen, 37075 Göttingen, Germany; 4 Department of Otorhinolaryngology, Head & Neck Surgery, University of Erlangen- Nuremberg, 91054 Erlangen, Germany; 5 Center of Hearing and Speech MEDINCUS, Clinical Trials Department, Kajetany, Poland; 6 Institute of Sensory Organs, Kajetany, Poland; 7 World Hearing Center of the Institute of Physiology and Pathology of Hearing, Kajetany, Poland; 8 Department of Teleaudiology and Screening, World Hearing Center, Institute of Physiology and Pathology of Hearing, 10 Mochnackiego Street, Warsaw, 02-042 Poland; 9 Department of Otolaryngology, Hannover Medical School, 30625 Hannover, Germany; 10 Cluster of Excellence Hearing4all, Medical University Hannover, 30625 Hannover, Germany; 11 Zentrum fuer Mittelohrchirurgie (Centre for Middle Ear Surgery), ENT Practice, Brechtener Straße 59, 44536 Luenen, Germany; 12 Department of Otorhinolaryngology, Head & Neck Surgery, University Clinic St. Poelten, St. Pölten, 3100 Austria; 13 Department of Otorhinolaryngology, Head and Neck Surgery, 1030 Wiener Gesundheitsverbund, Klinik Landstraße, Vienna, 1030 Austria; 14 Sigmund Freud Private University, Vienna, 1020 Austria; 15 Department of Otorhinolaryngology, Head and Neck Surgery, Kepler University Hospital GmbH, Krankenhausstrasse 9, Linz, 4020 Austria; 16 Medical Faculty, Johannes Kepler University Linz, Altenbergerstrasse 69, Linz, 4040 Austria; 17 Department of Otorhinolaryngology, Medical University of Vienna, Vienna, 1090 Austria

**Keywords:** Partial ossicular replacement prostheses, MXACT partial prosthesis, MXACT PRO partial prosthesis, MCLIP partial prosthesis, MCLIP ARC partial prosthesis

## Abstract

**Purpose:**

This multicentric, retrospective study aimed to analyze the safety and effectiveness of the mXACT Partial Prosthesis, mXACT PRO Partial Prosthesis, mCLIP Partial Prosthesis, and mCLIP ARC Partial Prosthesis.

**Methods:**

Patients underwent partial ossicular replacement with a mXACT Partial Prosthesis, mXACT PRO Partial Prosthesis, mCLIP Partial Prosthesis, or mCLIP ARC Partial Prosthesis (MED-EL). The clinical data was retrospectively analyzed according to the coupling type, bell or clip. Follow-up examination included access to medical records (for adverse events (AE) of the patients, ear microscopy and pure-tone audiometry to determine the post-operative pure tone average of the frequencies 0.5, 1, 2 and 3 kHz (PTA_4_)). The post-operative PTA_4_ air bone gap (ABG) was used to evaluate the audiological outcome. A post-operative PTA_4_ ABG ≤ 20 dB was defined as successful rehabilitation. A post-operative minimum and maximum follow-up period was not defined.

**Results:**

203 patients were implanted, 202 patients were examined for AEs (bell group: 68 patients, clip group: 134 patients), 186 patients were examined audiologically (bell group: 63 patients, clip group: 123 patients). In the bell group, 4 patients (5.9%) had 4 AE (thereof 2 revision surgeries and 2 dislocations/extrusions), and in the clip group 15 patients (11.2%) had 28 AEs (thereof 2 revision surgeries and 1 dislocation/extrusion). A post-operative PTA_4_ ABG of ≤ 20 dB and therefore successful rehabilitation was reached by 44 patients (69.8%) in the bell, and by 89 patients (72.4%) in the clip group. The mean post-operative PTA_4_ ABG was 17.8 ± 10.2 dB for patients implanted with a bell prosthesis and 16.3 ± 10.1 dB for patients implanted with a clip prosthesis. Individual bone conduction (BC) PTA_4_ thresholds were stable in 60 patients (95.2%) implanted with a bell prosthesis and 120 patients (97.6%) implanted with a bell prosthesis.

**Conclusion:**

Clinical data demonstrated satisfactory short-term audiological results after implantation of the mXACT Partial Prosthesis, mXACT PRO Partial Prosthesis, mCLIP Partial Prosthesis, and mCLIP ARC Partial Prosthesis in the majority of patients. The partial ossicular replacement prostheses are safe and effective.

## Introduction

The purpose of middle ear surgery is to create a dry ear by removing underlying pathologies and preventing recurrence. Ossiculoplasty can restore proper function of the ossicular chain, and titanium ossicular replacement prostheses can be an effective tool in this surgery. First appearing in Germany in 1994 [1], these prostheses serve to restore sound transmission between the tympanic membrane and the stapes footplate. Partial ossicular replacement prostheses (PORP) can be used if at least the stapes and its footplate are still functional; other cases may require total ossicular replacement prostheses (TORP) if only the footplate is present and functional. Various materials have been used for PORPs, including metals (titanium, platinum, gold), plastics (polyamide, polyethylene), Teflon^®^, and ceramics (hydroxyapatite, oxide ceramic, carbon, calcium phosphate ceramic, glass ceramic) [2]. The use of titanium has certain advantages such as its lightweight, good biocompatibility [3] and MRI compatibility at 1.5 and 3.0 and 7.0 tesla [4], and functional results with titanium PORPs have been good [5].

Ossiculoplasty strives to close the air-bone gap (ABG) in patients who have malformations of the ossicular chain, trauma or various pathologies such as chronic otitis media (COM) or cholesteatoma. This is achieved by re-establishing a connection between the tympanic membrane and the oval window, for example, with a prosthesis [6–10]. The aim of this multicenter study was to confirm the short-term safety and performance of the mXACT Partial Prosthesis, mXACT PRO Partial Prosthesis, mCLIP Partial Prosthesis and mCLIP ARC Partial Prosthesis (MED-EL, Innsbruck, Austria) as treatment for CHL and MHL.

## Materials and methods

### Device design

The titanium PORPs in this study were developed by MED-EL (MED-EL, Innsbruck, Austria) and first implanted in 2021. The mXACT Partial, mXACT PRO Partial, mCLIP Partial and mCLIP ARC Partial prostheses (Table 1) are made of medical-grade titanium and consist of a headplate and shaft, as well as a structure to couple the prosthesis to the stapes head. The shaft and coupling mechanism of the mXACT Partial and mXACT PRO Partial prostheses (bell prostheses) both contain an open filigree headplate; the difference is that the PRO variant has a trimmable, adjustable length between 0.75 and 3.5 mm. The mCLIP Partial and mCLIP ARC Partial prostheses (clip prostheses, available in 10 functional length variants: 0.75, 1.0, 1.25, 1.5, 1.75, 2.0, 2.25, 2.5, 3.0, 3.5 mm) use a clip design for coupling to the stapes head; the mCLIP ARC Partial additionally has a ball joint that allows for rotation [11].

**Table 1:**
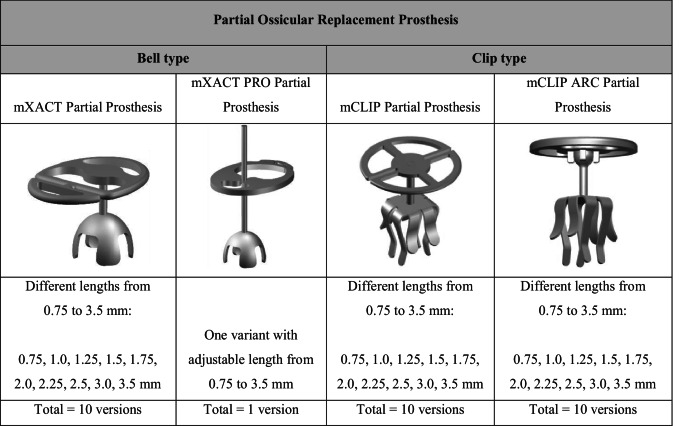
Tympanoplasty Prostheses. The functional length of the MED-EL partial prostheses is defined as the distance between the tympanic membrane reconstruction and the stapes head

### Ethical considerations

This study was conducted in Germany, Austria and Poland in agreement with the Declaration of Helsinki and was approved by the relevant German, Austrian and Polish ethics committee(s) (Göttingen: 1/9/20; Hannover: 9456_BO_S_2020; Erlangen: 456_20 Bc; Lünen: 2020-829-b-S; Freiburg: 22–1142-retro; Linz: 1257/2022; Wels: 1257/2022; Gesundheitsverbund, Klinik Landstraße: EK_23_005_XX; AKH Vienna: 2296/2021; Sankt Pölten: GS1-EK-4/777–2022; Warsaw: Oświadczenie nr. 10/2023r.) as a post-market clinical follow-up study.

### Study design

This retrospective, multicenter study included 203 patients implanted with one of four different PORPs between January 2021 and December 2022. It was conducted across 11 study sites: 5 study sites in Germany (Göttingen, Hannover, Erlangen, Lünen, Freiburg), 1 study site in Poland (Kajetany) and 5 study sites in Austria (Linz, Wels, Gesundheitsverbund Wien, AKH Wien, St. Pölten). Inclusion criteria were patients of all ages implanted (initial implantation or revision implantation) with an mXACT Partial Prosthesis, mXACT PRO Partial Prosthesis, mCLIP Partial Prosthesis or mCLIP ARC Partial Prosthesis (MED-EL, Innsbruck, Austria) between January 2021 and December 2022 in the participating study centers. Adverse events were collected for all patients until December 2022; audiological performance with the device was only assessed for patients with complete audiological datasets.

186 out of 203 (91.6%) patients were included in the audiological assessment. 17 (8.4%) were excluded due to incomplete audiological datasets. 202 out of 203 were evaluated for AEs. One (0.5%) patient was excluded due to missing post-operative follow-up information.

### Follow-up time

All patients implanted with a MED-EL PORP by December 31, 2022 were included in the study and all adverse events were collected until that day. The median follow-up time for the collection of adverse events was 275 days (range: 0–666 days). Patients with audiological follow-ups (1 pre- and 1 post-operative audiological measurement) were analyzed to assess the prostheses’ performance. Routine audiological measurements were taken at different times for different patients and study sites (range: 3–302 days; median: 70 days). The audiological follow-up time was therefore shorter than for the AEs. PTA_4_ was calculated as a four-frequency mean of 0.5, 1, 2, and 3 kHz according to the American Academy of Otolaryngology-Head and Neck Surgery [12].

### Audiometric methods

#### PTA_4_ ABG

The ABG was calculated by audiometric testing of the post-operative bone conduction (BC) and air conduction (AC) thresholds at 0.5, 1, 2, and 3 kHz.

#### BC PTA_4_

The individual differences (Δ) between BC post-operative and pre-operative PTA_4_ were calculated to determine the safety of the implantation procedure.

### Adverse events

Surgical, procedure- and device-related AEs in the operated ear that occurred intra- and post-operatively were collected.

### General information

PTA_4_ ABG and AEs were analyzed descriptively. BC PTA_4_ was calculated inferentially. Graphs were created and statistical analysis was performed with GraphPad Prism 7.04 (GraphPad Software, Inc.).

## Results

### Demographics

202 patients including 19 children were examined for AEs, and 186 patients who had complete audiological datasets were examined audiologically. The patients were retrospectively allocated into two groups based on the coupling type, bell or clip. *Bell group*: 92.6% of patients (63/68) had complete audiological datasets (mXACT Partial Prosthesis 48/52 patients, mXACT PRO Partial Prosthesis 15/16 patients). In the audiological cohort the mean age was 40.5 ± 17.0 years (41.0 ± 17.4 years in the AE cohort), 46.0% were female (44.1% in the AE cohort), and 46.0% were implanted on the right side (45.6% in the AE cohort). The CHL/MHL ratio was 31.7/68.3% in the audiological and 35.3/64.7% in the AE cohort.

Clip group: 91.8% of patients (123/134) had complete audiological datasets (mCLIP Partial Prosthesis 68/72 patients, mCLIP ARC Partial Prosthesis 55/62 patients). In the audiological cohort the mean age was 45.6 ± 19.2 years (46.3 ± 19.2 years in the AE cohort), 48.8% were female (50.7% in the AE cohort), and 54.5% were implanted on the right side (56.7% in the AE cohort). The CHL/MHL ratio was 30.1/69.9% in the audiological and 29.1/70.9% in the AE cohort. Detailed demographic information is provided in Table 2.

**Table 2:** Demographics. F: female; m: male; SD: standard deviation; R: right; l: left; HL: hearing loss; CHL: conductive hearing loss; MHL: mixed hearing loss

	BELL GROUP		CLIP GROUP		All partial prostheses (pediatrics)
	mXACT Partial	mXACT PRO Partial	mCLIP Partial	mCLIP ARC Partial	
N (audiological cohort), thereof children	48 (4)	15 (3)	68 (6)	55 (6)	19 (19)
Gender (f/m)	22/26	7/8	33/35	27/28	8/11
Age in years: mean ± SD range	42.5 ± 16.7 8–71	34.1 ± 17.2 8–61	44.8 ± 19.1 9–81	46.5 ± 19.5 5–79	11.5 ± 3.1 5–16
Implantation side (r/l)	25/23	3/12	34/34	33/22	6/13
Type of HL (CHL/MHL)	14/34	6/9	24/44	13/42	8/11
N (AE cohort), thereof children	52 (4)	16 (3)	72 (6)	62 (6)	19 (19)
Gender (f/m)	23/29	7/9	36/36	32/30	8/11
Age in years: mean ± SD range	42.7 ± 17.1 8–79	35.4 ± 17.4 8–61	45.4 ± 18.8 9–81	47.3 ± 19.8 5–84	11.5 ± 3.1 5–16
Implantation side (r/l)	28/24	3/13	38/34	38/24	6/13
Type of HL (CHL/MHL)	18/34	6/10	25/47	14/48	8/11

### Audiometric results

**Fig. 1:**
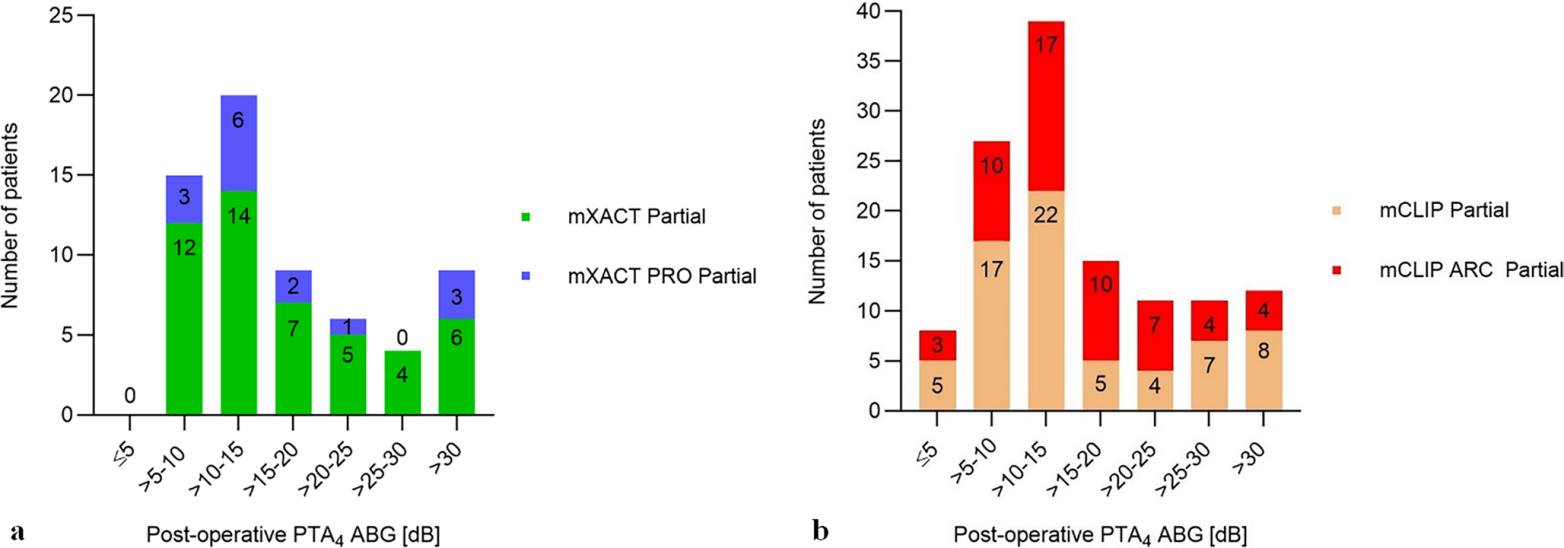
**Success rates in 5 dB bins.** a) Bell group: mXACT Partial Prosthesis 48 patients (green), mXACT PRO Partial Prosthesis 15 patients (blue). Clip group: mCLIP Partial Prosthesis 68 patients (salmon), mCLIP ARC Partial Prosthesis 55 patients (red)

All patients (*n* = 186):

Bell group: 44 patients (44/63 = 69.8%) had a PTA_4_ ABG of ≤ 20 dB (95% confidence interval (CI): 58.5%, 81.1%) and therefore a successful outcome (Fig. 1a). The mean post-operative PTA_4_ ABG was 17.8 ± 10.2 dB (Table 3). The median post-operative follow-up time was 91.0 days (mean: 97.4 ± 62.1 days (range: 15 to 294 days)).

**Table 3:** Audiology and Safety. F/U: follow-up; SD: standard deviation; PTA_4_ ABG: pure tone average air bone gap; HL: hearing loss; BC: bone conduction; AE: adverse events

	BELL GROUP		CLIP GROUP		
	mXACT Partial	mXACT PRO Partial	mCLIP Partial	mCLIP ARC Partial	All partial prostheses (pediatrics)
Audiology					
F/U in days: median/mean ±SD range	73.5/85.0 ± 57.9 17–237	105.0/123.5 ± 77.3 21–294	78.5/77.6 ± 46.0 3–302	28.0/57.5 ± 50.6 13–223	105.0/102.6 ± 70.9 17–263
Post-operative PTA_4_ ABG [dB] Mean ± SD	17.8 ± 10.3	17.9 ± 10.0	16.3 ± 10.9	16.5 ± 9.2	12.3 ± 5.7
PTA_4_ ABG ≤ 20 dB N (%) patients	33/48 68.8%	11/15 73.3%	49/68 72.1%	40/55 72.7%	17/19 89.5%
Pre-operative BC PTA_4_ [dB HL] Mean ± SD	18.5 ± 7.6	22.2 ± 9.1	21.4 ± 13.8	18.2 ± 13.8	10.8 ± 6.2
post-operative BC PTA_4_ [dB HL] Mean ± SD	18.0 ± 8.5	19.8 ± 8.4	18.9 ± 13.9	15.9 ± 13.6	9.5 ± 7.2
BC PTA_4_ deterioration > 10 dB HL N (%) patients	3/48 6.3%	0/15 0.0%	1/68 1.5%	2/55 3.6%	0/19 0.0%
Safety					
F/U in days: median/mean ±SD range	165.5/262.2 ±198.5 0–634	182.0/199.2 ± 106.5 65–432	374.5/368.1 ±177.2 0–666	205.0/221.8 ± 130.8 24–459	304.0/336.6 ± 211.8 64–634
N (%) patients with an AE Number of AEs + F/U AEs	2/52 (3.9%) 2 AEs + 0 F/U	2/16 (12.5%) 2 AEs + 0 F/U	13/72 (18.1%) 25 AEs + 2 F/U	2/62 (3.2%) 3 AEs + 0 F/U	0
- thereof revision surgeries	1/52 (1.9%)	1/16 (6.3%)	1/72 (1.4%)	1/62 (1.6%)	0
- thereof device dislocations/extrusions	1/52 (1.9%)	1/16 (6.3%)	1/72 (1.4%)	0	0

**Fig. 2:**
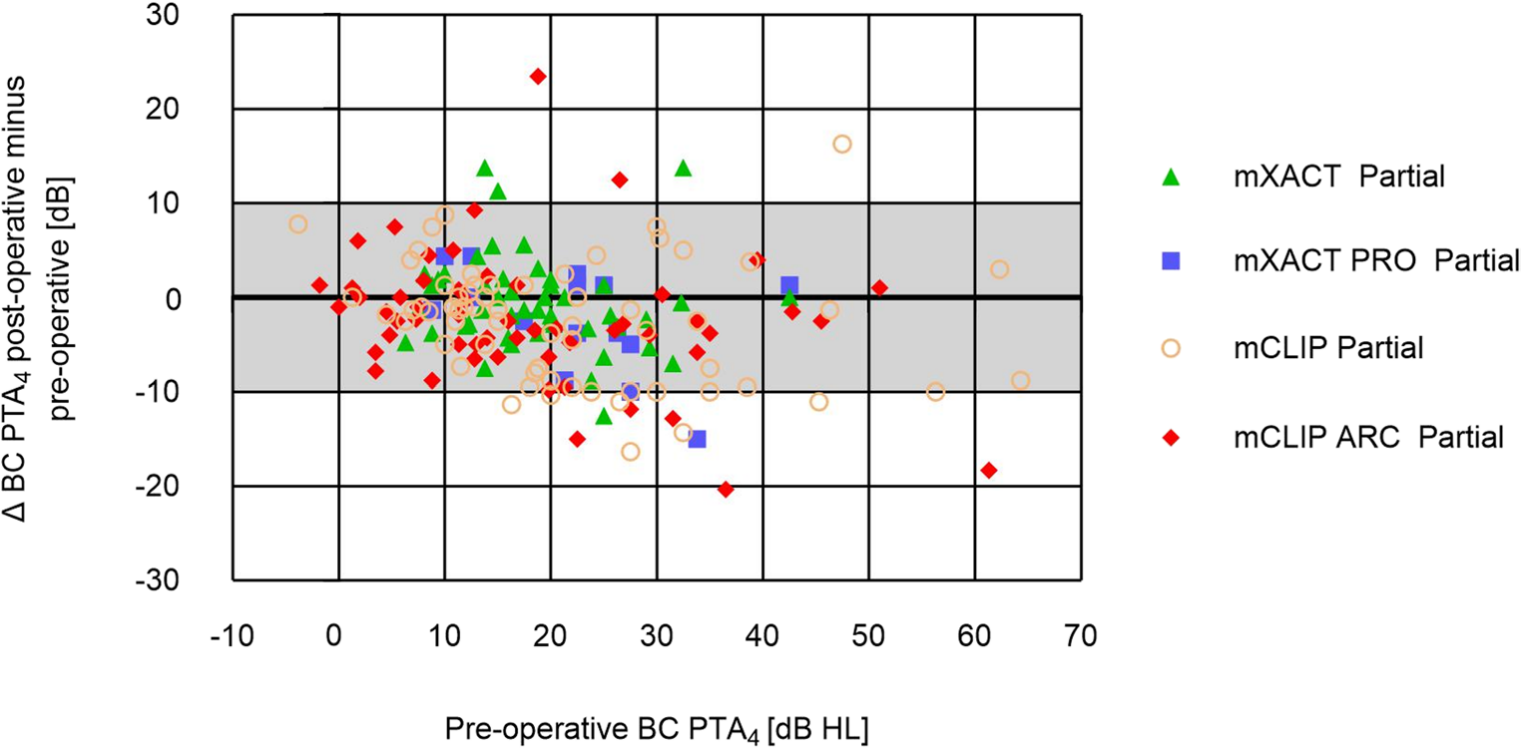
**Preoperative BC PTA**_**4**_ **compared to post-operative minus pre-operative BC PTA**_**4**_ **(Δ).** Grey painted area: patients with BC PTA_4_ differences inside the test-retest-variability. Patients above the grey painted area have a post-operative BC PTA_4_ deterioration of ≥ 10 dB HL. Patients below the grey painted area have a post-operative BC PTA_4_ improvement of ≥ 10 dB HL. mXACT Partial Prosthesis (green triangles), mXACT PRO Partial Prosthesis (blue squares), mCLIP Partial Prosthesis (salmon circles), mCLIP ARC Partial Prosthesis (red diamonds)

Clip group: 89 patients (89/123 = 72.4%) had a PTA_4_ ABG of ≤ 20 dB (95% CI: 64.5%, 80.3%) and therefore a successful outcome (Fig. 2b). The mean post-operative PTA_4_ ABG was 16.3 ± 10.1 dB (Table 3). The median post-operative follow-up time was 61.0 days (mean: 68.6 ± 49.1 days (range: 3 to 302 days)). The postoperative PTA_4_ ABG was not significantly different between the two groups (Kolmogorov-Smirnov test, *p* = 0.5326), and also not between the individual four variants (Kruskal-Wallis test, *p* = 0.6023).

Pediatrics: Of the patients below 18 years of age (mXACT Partial 4 patients, mXACT PRO Partial 3 patients, mCLIP Partial 6 patients, mCLIP ARC Partial 6 patients), 17 (17/19 = 89.5%) had a PTA_4_ ABG of ≤ 20 dB (95% CI: 75.7%, 100%) and therefore a successful outcome. The mean post-operative PTA_4_ ABG was 12.3 ± 5.7 dB (Table 3). The median post-operative follow-up time was 105.0 days (mean: 102.6 ± 70.9 days (range: 17 to 263 days)). The postoperative PTA_4_ ABG was not significantly different between adults and children (Kolmogorov-Smirnov test, *p* = 0.1529).

### Safety

Bell group: The mean BC PTA_4_ threshold was 19.4 ± 8.1 dB HL pre-operatively, and 18.5 ± 8.5 dB HL post-operatively. The individual Δ PTA_4_ BC thresholds were stable in 60 (60/63 = 95.2%) patients and within the fluctuation range of ± 5 dB HL. Three (3/63 = 4.8%) patients implanted with the mXACT Partial Prosthesis had a BC PTA_4_ deterioration of > 10 dB HL (range from 11.3 to 13.8 dB HL) after the implantation (Fig. 2). *Clip group*: The mean BC PTA_4_ threshold was 20.0 ± 13.9 dB HL pre-operatively, and 17.6 ± 13.8 dB HL post-operatively. The individual Δ PTA_4_ BC thresholds were stable in 120 (120/123 = 97.6%) patients and within the fluctuation range of ± 5 dB HL. Three (3/123 = 2.4%) patients, one implanted with the mCLIP Partial Prosthesis and two implanted with the mCLIP ARC Partial Prosthesis had a BC PTA_4_ deterioration of > 10 dB HL (range from 12.5 to 23.5 dB HL) after the implantation (Fig. 2). The Δ BC PTA_4_ was not significantly different between the two groups (Kolmogorov-Smirnov test, *p* = 0.2397), and also not between the individual four variants (Kruskal-Wallis test, *p* = 0.3099). *Pediatrics*: The mean BC PTA_4_ threshold was 10.8 ± 6.2 dB HL pre-operatively, and 9.5 ± 7.2 dB HL post-operatively. The individual Δ PTA_4_ BC thresholds were stable in all patients. The Δ BC PTA_4_ was not significantly different between adults and children (Kolmogorov-Smirnov test, *p* = 0.2648).

### Adverse events

To evaluate the safety of the partial ossicular replacement prostheses, all surgical-, procedure- and device-related events were retrospectively collected. All events that occurred were anticipated and known that they can occur after partial ossicular replacement procedures. Nevertheless, due to the retrospective study design, the status of some events is incomplete.

####  Bell group

4 patients (4/68 = 5.9%) had 4 AEs. One patient had a dislocated prosthesis (mXACT Partial) and an extirpation through the tympanic membrane 55 days post-operatively; a revision surgery was performed with a type IV tympanoplasty. Another patient implanted with a mXACT Partial had a taste disorder 61 days post-operatively, which resolved 117 days post-operatively. One patient implanted with the mXACT PRO Partial had a residual hearing reduction 92 days post-operatively. Prosthesis dislocation or sclerosis of the footplate was assumed, and a revision surgery was recommended. In another patient the chorda tympani was damaged at the day of implantation (mXACT PRO Partial; no further information available).

####  Clip group

15 patients (15/134 = 11.2%) had 28 AEs and 2 follow-up AEs. mCLIP Partial users (13 patients with 25 AEs and 2 follow-up AEs): One patient had a post-inflammatory meatal fibrosis 78 days post-operatively, and a revision surgery was suggested but the patient did not return to the clinic. One patient suffered from residual conductive hearing loss 101 days post-operatively, and the prosthesis was readjusted (no further information). One patient had cold symptoms with subsequent increased ear pressure and a reduction in hearing 336 days post-operatively, which was resolved with an antibiotic (Fentrinol) for nasal congestion. One patient suffered from deprivation due to the long-lasting hearing loss and had to use a hearing aid temporarily (it was unclear if this event occurred before or after mCLIP Partial implantation). One patient reported pressure and hearing reduction 13 days post-operatively, which resolved. One patient had a wet feeling in the ear and was discovered to have a polyp behind the tympanic membrane which resolved with suction and Otostop treatment. One patient suffered an otorrhea 65 days post-operatively which was treated with an antibiotic (Infectociprocort).

Several patients reported more than one AE. One patient had a superinfected radical cavity 90 days post-operatively which resolved with suction of the inflammation material and treatment with a corticoid (Betnesol); 189 days post-operatively this patient suffered from a mild radical cavity inflammation which was also resolved with suction of the inflammation material and treatment with an antibiotic (InfectoCipro). It is assumed that residual inflammation caused this follow-up AE. This patient had a second AE 413 days post-operatively: cerumen and malodorous material in the ear, which was resolved by antibiotic ear cleaning (Otanol). Another patient had two AEs: 28 days post-operatively, otitis externa mycotica, pain and ear secretion was reported, which resolved by ear cleaning and treatment with antimycotics (Clotrimazol ointment and Diflucan). 82 days post-operatively, this same patient reported a “rushing ear”, and a hearing aid was prescribed (no further information). Another patient had two AEs: 4 days post-operatively, a loss of taste occurred and was treated with Neurobion lozenges. This patient then suffered otitis media 18 days post-operatively, which resolved with antibiotics (Infectociprocort and Clavamox). One patient had three AEs: 13 days post-operatively bleeding out of the ear canal occurred, which resolved with Diprogenta ointment strips. The patient then suffered an infection-associated seromucotympanum 152 days post-operatively after a COVID-19 infection, which resolved with decongestant drops. Parallel to this, it was discovered that the cartilage graft between the prosthesis and tympanic membrane (to seal the prosthesis) was not in the optimal position anymore, causing the medial part of the prosthesis to be in direct contact with the tympanic membrane (there was no tympanic membrane perforation; protrusion of the prosthesis was suspected). One patient had four AEs and one follow-up AE: 5 days post-operatively, pain and dizziness was reported, which resolved with antibiotics. Then, 20 and 30 days post-operatively (AE and follow-up AE) the patient suffered from a radical cavity secretion which was resolved by cleaning the ear and applying Otostop, followed up with local antibiotic ointment (Aureocort) treatment. The third AE, ear secretion, was again resolved with Otostop. The fourth AE was a fungal infection of the radical cavity; parallel to this, the headplate of the mCLIP Partial Prosthesis migrated. The patient was treated with antibiotics (Clothrimoxazol), and an MRT appointment was recommended (no further information). One patient had five AEs: 20 days post-operatively the patient reported pain, which was resolved with medication and ear cleaning; 195 days post-operatively the patient reported pain again (otalgia; no further information for this AE); 281 days post-operatively wet ears were treated with nose drops (Betnesol-N); 349 days post-operatively the prosthesis extruded, and the patient noticed a reduction in hearing. The patient reported having wet ears again 425 days post-operatively, which was treated with an antibiotic (Otanol). mCLIP ARC Partial users (2 patients with 3 AEs): One patient had a parietal recurrent eardrum perforation 53 days post-operatively, which resolved with revision surgery. Another patient had two AEs: 4 days post-operatively the patient reported a loss in sense of taste, which was treated with antibiotics (Clavamox); 21 days post-operatively, an anterior/posterior perforation of the tympanic membrane occurred (no further information).

Additionally, two mCLIP Partial users had recurrent cholesteatoma, which is considered neither surgical-, procedure-, or device-related: one after a canal wall up type III tympanoplasty, which resolved 609 days post-operatively with a second look surgery, and another (14-year-old child) 353 days post-operatively (second look surgery was planned; no further information).

## Discussion

There are many aspects which could influence the choice of a prosthesis, even issues with country-specific funding or other clinical administration decisions. From a surgeon’s point of view, personal experience related to the anatomical condition of the middle ear cavity is a key factor. Another is the possibility to adjust a prosthesis to fit in the middle ear cavity in connection with the tympanic membrane without the need for large reductions of the posterior bone wall of the external meatus, potentially reducing complications. The mucosal status and amount of drainage of the middle ear, condition of the ossicular chain and presence of the posterior ear canal can also influence the outcome with ossiculoplasty [13, 14]. Having a wide range of PORP variations to choose from is helpful since every patient’s anatomy is unique and surgeons’ preferences also vary. For example, in smaller tympanic cavities, bell type PORPs such as the mXACT and mXACT PRO may work better than clip type PORPs since the bell coupling structure requires less space on the stapes head (height 1.1 mm vs. 1.6 mm). The mXACT PRO package includes a sizer kit for testing the individual needed length of the prosthesis and the prosthesis itself features an adjustable length, allowing the surgeon to fit it to different anatomies without requiring the clinic to stock multiple length variants. The two clip-type PORPs can be broken down into those with a ball joint (mCLIP ARC) and those without (mCLIP). The clip mechanism is intuitive, and it provides predictable stability with little adjustment required. The clip feet are sufficiently flexible to avoid excessive pressure on the stapes and footplate, while at the same time rigid enough to prevent deformation and to maintain the mechanical stability of the reconstruction. The curved design of the mCLIP ARC facilitates atraumatic placement even when visibility is restricted, and it is often possible to position the prosthesis with less manipulation, while still achieving a very stable coupling to the incus remnant, even in narrow tympanic cavities. Of course, as with any new implant, some familiarization and practice are initially required. The increased mobility of the prosthesis headplate because of the ball joint requires slightly different handling, but in return it provides improved visibility during placement and allows the headplate to adjust to movements of the tympanic membrane. The ball joint improves surgical handling by facilitating both the placement of the clip on the stapes head and the insertion of cartilage between the prosthesis and the tympanic membrane. Bevis et al. further proposes that the flexible ball joint may potentially reduce the risk of prosthesis migration [9].

### Post-operative PTA_4_ ABG

Middle ear reconstruction can be challenging, and success varies greatly among the published literature [3, 13, 15–17]. Success rates with a PORP are usually measured by the percentage of patients achieving a post-operative PTA_4_ ABG of ≤ 20 dB [15]. This study reported on results with the new MED-EL PORP, which have proven to be effective with 69.8% of patients implanted with a bell type prosthesis (mXACT Partial, mXACT PRO Partial) and 72.4% of patients implanted with a clip type prosthesis (mCLIP Partial, mCLIP ARC Partial) achieving a PTA_4_ ABG of ≤ 20 dB. The prostheses were also effective in the pediatric sub-analysis with 89.5% of children having a successful outcome, nevertheless it should be kept in mind that the sample size of the pediatric sub-cohort is relatively small. The audiological outcomes of the clip group patients in this study are comparable to those presented in several studies on the mCLIP Partial [7] and mCLIP ARC Partial [9, 18] PORP, as well as similar clip type PORP in earlier literature [19–21]. Similar audiological results can also be seen between the bell group patients in our study and those in previous reports of prostheses with a comparable design [19].

In a study by Quesnel et al. (2010), 53.8% of the children achieved a PTA_4_ ABG of ≤ 20 dB post-operatively (TTP-VARIO BELL Partial Prosthesis; *n* = 71; mean post-operative follow-up was 30 ± 17.7 months; PTA_4_ was calculated at 0.5, 1, 2 and 4 kHz). In our study, we have exceeded the findings of Quesnel by more than 35% points since 89.5% of the children (*n* = 19) achieved a PTA_4_ ABG of ≤ 20 dB post-operatively.

Our study aimed to achieve a post-operative PTA_4_ ABG of ≤ 20 dB in at least 53.8% patients implanted with the partial ossicular replacement prostheses; this goal was exceeded. Success rates ranging from 62.8% to 72.7% have been reported in five systematic reviews and meta-analyses [15, 21–24], which correlates well with the results in this study.

Bevis et al. reported on 31 patients implanted with the mCLIP ARC Partial prosthesis, with 63% achieving a post-operative PTA_4_ ABG of ≤ 20 dB at short-term follow-up and 77% at mid-term follow-up [9]. A multicentric study on the mCLIP ARC was able to show a PTA_4_ ABG of 72.7% in 55 patients. Rasse et al. [7] reported on the first clinical results with the mCLIP Partial Prosthesis, in which 49/68 patients (72.1%) achieved a post-operative PTA_4_ ABG of ≤ 20 dB.

Schmerber et al. [19] retrospectively evaluated 61 patients with a Kurz titanium Bell partial ossicular replacement prosthesis, with 77% of the patients reaching a PTA_4_ ABG ≤ 20 dB 3 months after the surgery. Kahue et al. [20] reported on 130 patients implanted with a CliP partial titanium ossicular prosthesis (Kurz, Dusslingen, Germany). The follow-up period was 4 months and >12 months (mean: 42 ± 17 months). 63% of the 120 patients followed up for 4 months achieved a PTA_4_ ABG of ≤ 20 dB. Bartel et al. [21] evaluated 14 studies with 441 PORP patients. 71.3% of the patients had an ABG closure of ≤ 20 dB post-operatively. The post-operative follow-up time was not provided.

Device safety was assessed by collecting information on adverse events, including changes in BC thresholds, as well as device displacement and extrusion. Mucosa and aeration of the middle ear significantly contribute to the success of ossiculoplasty. In poorly ventilated middle ears, the risk for resorption of bioactive materials or extrusion of bioinert materials is increased [16].

### Adverse events

Nineteen (19/202 = 9.4%) of the 202 patients reported 32 AEs and 2 follow-up AE. None of the patients who reported AEs had PTA_4_ BC threshold shifts > 10 dB HL. However, 9 patients who reported AEs experienced post-operative PTA_4_ ABG > 20 dB (range: 21.3–63.3 dB), which means that these patients did not benefit audiologically. Six of these 9 patients did have a post-operative ABG between 21 and 30 dB and may have still benefitted from the surgery depending on their initial preoperative ABG which was unfortunately unknown. One limitation of the study lies in the lack of pre-operative ABG data.

More adverse events were reported in the clip group (15 patients with 28 AEs and 2 follow-up AEs), as compared to the bell group (4 patients with 4 AEs), even though there were only double the number of patients within the groups (*n* = 134 vs. *n* = 68). Nevertheless, the median follow-up was longer for the clip than the bell group (median 296 vs. 169 days). Additionally, within the clip group 10 AEs could be resolved by standard antibiotic therapy, 3 AEs with conventional Otostop treatment, and 2 AEs with corticoid therapy (Betnesol). These can be considered minor events. Taste disorders occurred in a similar frequency in both groups (~ 1.5% of patients). Interestingly, revision surgery rates and dislocation/extrusion rates (see Table 3) were both slightly lower in the clip group. Importantly, in both groups all events were anticipated and known side effects of otologic surgical procedures.

### PTA_4_ BC thresholds

PTA_4_ BC thresholds were stable in 180 (180/186 = 96.8%) patients and within the fluctuation range of ± 5 dB HL. The mean BC PTA_4_ threshold was 19.8 ± 12.2 dB HL pre-operatively, and 17.9 ± 12.2 dB HL post-operatively.

*Six* (6/186 = 3.2%) patients, three in the bell and three in the clip groups, had a BC PTA_4_ deterioration of > 10 dB HL (range: 11.3 to 23.5 dB HL) post-operatively.

One of the patients implanted with the mCLIP ARC Partial (Chronic otitis media/erosion of the incus) had a post-operative PTA_4_ ABG of 12.8 dB and a PTA_4_ BC threshold deterioration of 12.5 dB HL. These results confirm a minor deterioration of the PTA_4_ BC but also show an audiological benefit for the patient. One patient implanted with the mXACT Partial (cholesteatoma) had a minor PTA_4_ BC threshold deterioration of 13.8 dB HL and a PTA_4_ ABG of 47.5 dB. One patient using the mCLIP Partial (Chronic mastoiditis/Otoliquorrhoe/Meningoencephalocele) and one patient with the mXACT Partial (chronic otitis media) had minor PTA_4_ BC threshold deteriorations of 16.3 dB HL and 11.3 dB HL, respectively; the PTA_4_ ABGs were < 20 dB (17.5 dB and 15.0 dB, respectively), which again showed that they benefitted from their protheses. One patient (mCLIP Partial) (cholesteatoma) had a minor PTA_4_ BC threshold deterioration of 13.8 dB HL and a PTA_4_ ABG of 23.8 dB. One patient with the mXACT Partial (cholesteatoma) had a PTA_4_ BC threshold deterioration of 23.5 dB HL and a PTA_4_ ABG of 47.5 dB, which indicates an ongoing hearing problem.

### Extrusion/displacement rates

Our study had 3 device displacements or extrusion. This amounts to a displacement/extrusion rate of 2.9% (2/68 patients) in the bell group and 0.75% (1/134 patients) in the clip group, both of which correlate well with displacement and extrusion rates reported in similar studies.

Lahlou et al. [17] found a displacement rate of 1.8% (3/163 patients) corrected by revision surgery and an extrusion rate of 3% (5/163 patients). Two additional revision surgeries were reported: 1 case with retraction pocket in the attic region without hearing impairment (retraction supported by additional cartilage palisade), in the other case the reason for revision surgery was not specified. Birk et al. [25] found an extrusion rate of 0.4% (1/226 patients). Gostian et al. [10] reported a displacement rate of 2.1% (1/47 patients). In a systematic review consisting of five studies including 99 PORP recipients, Omar found an extrusion rate of 3% [15].

Dislocation rates have been found to increase with post-operative time, while extrusions appear to happen shortly following the surgery [17]. Many patients who undergo ossiculoplasty have ears with underlying pathologies, such as cholesteatoma. Recurrences of these underlying pathologies or a suboptimal prosthesis size can lead to dislocation [26, 27]. In this study, two patients implanted with the mCLIP Partial were affected with cholesteatoma recurrence several months after implantation, which necessitated second-look surgeries. However, recurrence of underlying pathologies cannot be attributed to the usage of a prosthesis. Since a suboptimal prosthesis length can also lead to prosthesis dislocations, the use of intraoperative sizing tools is recommended in general. Nevertheless, the relatively low dislocation and extrusion rates found in this study merit longer-term follow-up, but the results have so far been encouraging.

### Study limitations

One important limitation of this study is the relatively short and quite diverse audiological follow-up and the absence of pre-operative AC PTA_4_ audiological data. Nevertheless, this study’s reporting of the success rate (rate of patients with a post-operative PTA_4_ ABG within 20 dB) alone has also been seen in several comparable publications and is widely accepted in the field. In addition, the safety follow-up (AE collection) was longer than the audiological follow-up and also varies quite a bit within the groups, with a longer follow-up for the clip as compared to the bell group. These limitations must be considered, especially regarding adverse events.

## Conclusion

The patients in this study implanted with the MED-EL partial ossicular replacement prostheses achieved a mean post-operative PTA_4_ ABG of 16.9 ± 10.1 dB (*n* = 186); the success rate was slightly higher in the clip group (72.4%) as compared to the bell group (69.8%). The individual Δ PTA_4_ BC thresholds were stable in 96.8% of patients. Fewer AEs were reported in the bell group than the clip group; nevertheless, the need for revision surgeries and the rate of device dislocations or prosthesis extrusions was similar among the groups. Revision surgery and dislocation/extrusion rates were low for both groups at 2.9%/2.9% in the bell group and 1.5%/0.75% in the clip group. The audiological and safety results confirm that the passive middle ear implants are safe and effective and provide substantial benefit.

## Declarations

### Trial registration number

NCT05565339 (clinicaltrials.gov).
